# Vulnerability to omega-3 deprivation in a mouse model of NMDA receptor hypofunction

**DOI:** 10.1038/s41537-017-0014-8

**Published:** 2017-03-22

**Authors:** Rehnuma Islam, Marc-Olivier Trépanier, Marija Milenkovic, Wendy Horsfall, Ali Salahpour, Richard P. Bazinet, Amy J. Ramsey

**Affiliations:** 1 Department of Physiology, University of Toronto, Toronto, Canada; 2 Department of Nutritional Sciences, University of Toronto, Toronto, Canada; 3 Department of Pharmacology and Toxicology, University of Toronto, Toronto, Canada

## Abstract

Several studies have found decreased levels of ω-3 polyunsaturated fatty acids in the brain and blood of schizophrenia patients. Furthermore, dietary ω-3 supplements may improve schizophrenia symptoms and delay the onset of first-episode psychosis. We used an animal model of NMDA receptor hypofunction, NR1KD mice, to understand whether changes in glutamate neurotransmission could lead to changes in brain and serum fatty acids. We further asked whether dietary manipulations of ω-3, either depletion or supplementation, would affect schizophrenia-relevant behaviors of NR1KD mice. We discovered that NR1KD mice have elevated brain levels of ω-6 fatty acids regardless of their diet. While ω-3 supplementation did not improve any of the NR1KD behavioral abnormalities, ω-3 depletion exacerbated their deficits in executive function. Omega-3 depletion also caused extreme mortality among male mutant mice, with 75% mortality rate by 12 weeks of age. Our studies show that alterations in NMDAR function alter serum and brain lipid composition and make the brain more vulnerable to dietary ω-3 deprivation.

## Glutamate neurotransmission: A weakness for omega-3

Depletion of omega-3 fatty acids in a mouse model of schizophrenia with altered glutamate transmission has a lethal effect in males. Previous studies have suggested that omega-3 supplements may improve the symptoms of schizophrenia. Amy Ramsey and colleagues at the University of Toronto, Canada, show in an established genetic mouse model of the disease that omega-3 dietary supplementation increased brain omega-3 levels, but did not have any beneficial effects on features that mirror symptoms of patients with schizophrenia such as increased locomotor activity or reduced social behavior. Interestingly, omega-3 dietary depletion worsened the cognitive performance and drastically increased the mortality rate of male mutant mice. The mechanisms responsible for these effects remain to be determined, but the findings highlight a potentially serious vulnerability of patients to dietary omega-3 deficits.

## Introduction

Polyunsaturated fatty acids (PUFAs) make up a majority of the fat composition in the brain, acting as important regulators of cell membrane fluidity, mediators of cellular signaling and initiators of gene transcription.^1^ Given their role in neuron function, inadequate levels of PUFAs in the brain can affect signaling in neurons. In patients with schizophrenia and other brain disorders, reductions in PUFA concentrations have been detected in red blood cell membranes and post-mortem brain tissue.^2–7^ Furthermore, numerous clinical studies have shown that ω-3 supplementation provides beneficial effects, both as an antipsychotic adjunct therapy and as a monotherapy.^8, 9^ In particular, Amminger *et al*. ^10^ found that ω-3 supplementation had preventative effects on the progression to first-episode psychosis, and improved positive, negative and cognitive symptoms. However, it is not clear whether ω-3 supplements simply correct a diet that is low in PUFAs, or whether altered neurotransmission in schizophrenia could in fact cause a reduction in ω-3 PUFAs. It is also not known whether supranormal levels of ω-3 PUFAs would have additional therapeutic benefit.

We addressed these questions using a genetic mouse model of schizophrenia that has reduced levels of NMDA receptors. NR1 knockdown (NR1KD) mice express 10% of the normal levels of NMDA receptors due to a mutation in the gene that encodes the NR1 subunit (Grin1, GluN1).^11^ The NR1KD mouse was used to assess the effects of dietary ω-3 manipulations because it models a state of NMDAR hypofunction that is thought to occur in at least some forms of schizophrenia.^12^ Mice with NMDAR hypofunction also display behavioral endophenotypes of schizophrenia, including: hyperlocomotion and increased stereotypy, reduced sociability, impaired sensorimotor gating, impaired working memory, and impaired executive function.^11, 13^ The observed deficits in sociability and working memory are sexually dimorphic, with male mutant mice having an earlier onset and greater severity than females.^13^

Our study tested whether NR1KD mice were more vulnerable than wild-type mice to ω-3 deprivation, and whether supplementation could improve their phenotype. We used a diet protocol that was shown to reduce brain docosahexaenoic acid (DHA) concentrations to a similar extent as is observed in post-mortem brains of schizophrenia patients and those with related mental disorders.^14^ Since the brain is resistant to ω-3 depletion,^15–18^ we maintained both the breeder pairs and the experimental mice on one of three diets: standard mouse chow (control diet), ω-3-deficient chow, and ω-3 supplemented chow. We assessed adult mice with behavioral tests and subsequently performed fatty-acid analysis of serum and brain tissue. We discovered that NMDAR hypofunction alters brain and serum levels of PUFAs, increasing specific ω-6 and decreasing specific ω-3 and ω-9 fatty acids. Genetic reduction in NMDAR signaling causes a heightened vulnerability to low levels of dietary ω-3.

## Results

### NMDA receptor deficiency alters serum and brain fatty-acid composition

Even on the standard, control diet, NR1KD mice had basal differences in their brain levels of ω-3 and ω-6 fatty acids (Table 1). Notably, serum profiles did not always reflect brain fatty-acid composition. The omega-3 fatty-acid ALA (α-linolenic acid) was increased in NR1KD brains (NR1:0.066 ± 0.02, WT:0.038 ± 0.03) (*F*_(1,44)_ = 4.50, *p* < 0.05). The ω-6 fatty-acid LA (linoleic acid) was also increased in NR1KD brains (NR1:0.78 ± 0.05, WT:0.67 ± 0.09) (*F*_(1,44)_ = 10.59, *p* < 0.01). These alterations were absent in the serum. We detected decreases in NR1KD serum levels of gondoic ω-9 PUFAs, which was unchanged in the mutant brain.

**Table 1:** Brain fatty-acid profile of WT and NR1KD mice on experimental diets

	Control diet		Omega-3 deficient		Omega-3 rich	
	WT	NR1	WT	NR1	WT	NR1
Omega-3 fatty acids						
α-linolenic acid (ALA)	0.038 ± 0.034	**0.066** ^**†**^ ± 0.018	0.042 ± 0.024	0.032^**‡**^ ± 0.030	0.050 ± 0.024	0.038 ± 0.025
Eicosapentaenoic acid (EPA)	0.067 ± 0.043	0.039 ± 0.288	0.040 ± 0.022	0.041 ± 0.022	0.107^‡^ ± 0.043	**0.136** ^**‡**^ ± 0.027
Docosapentaenoic acid (DPA)	0.631 ± 0.110	0.649 ± 0.127	0.597 ± 0.116	0.677 ± 0.174	0.639 ± 0.070	0.653 ± 0.108
Docosahexaenoic acid (DHA)	14.104 ± 0.204	14.181 ± 0.601	6.925^**‡**^ ± 1.561	6.287^**‡**^ ± 0.897	14.962 ± 0.865	14.720 ± 0.776
Omega-6 fatty acids						
Linoleic acid (LA)	0.672 ± 0.088	**0.781** ^**†**^ ± 0.052	0.840^‡^ ± 0.045	**0.974** ^**†‡**^ ± 0.060	0.871^‡^ ± 0.078	0.925^‡^ ± 0.062
Eicosadienoic acid (EDA)	0.300 ± 0.021	0.348 ± 0.057	0.416^‡^ ± 0.037	0.440 ± 0.065	0.393 ± 0.105	0.411 ± 0.114
Dihomo-gamma-linolenic acid (DGLA)	0.413 ± 0.180	0.494 ± 0.167	0.435 ± 0.202	0.405 ± 0.212	0.626 ± 0.153	0.668 ± 0.108
Arachidonic acid (AA)	7.454 ± 0.201	7.528 ± 0.243	8.205^‡^ ± 0.450	8.241^‡^ ± 0.255	6.795^‡^ ± 0.238	**7.053** ^**‡**^ ± 0.297
Adrenic acid	2.721 ± 0.100	2.785 ± 0.190	3.649^‡^ ± 0.257	3.674^‡^ ± 0.105	2.301^‡^ ± 0.116	**2.253** ^**‡**^ ± 0.051
Osbond acid	0.300 ± 0.047	0.319 ± 0.058	6.997^**‡**^ ± 1.322	**7.837** ^**†‡**^ ± 0.673	0.163 ± 0.028	0.152 ± 0.009
Omega-9 fatty acids						
Oleic acid	18.646 ± 0.951	18.027 ± 0.466	17.088 ± 1.038	16.974 ± 1.005	18.763 ± 0.404	18.541 ± 0.784
Gondoic acid	2.344 ± 0.042	2.168 ± 0.100	2.161 ± 0.148	2.023 ± 0.180	2.344 ± 0.126	2.689 ± 1.000
Nervonic acid	1.119 ± 0.205	1.053 ± 0.212	1.173 ± 0.191	1.183 ± 0.141	1.172 ± 0.163	**0.954** ^**†**^ ± 0.213

Data includes males and females aged 10–15 weeks (Control diet (*n*=): WT 4M, 4F; NR1 5M, 5F. Deficient diet (*n*=): WT 5M, 5F; NR1 5M, 5F. Supplemented diet (*n*=): WT 5M, 5F; NR1 5M, 4F). Concentrations of LA were higher in NR1KD mice compared to WT across all diets. Omega-3-deficient diets increased omega-6 fatty-acid levels, while omega-3-rich diets reduced omega-6 fatty-acid levels. NR1KD mice displayed deficits in omega-9 fatty acids in both omega-3-deficient and rich diets. †*p* < 0.05 post-hoc comparison to wild-type on the same diet, ‡*p* < 0.05 post-hoc comparison to control diet within the same genotype. Data expressed as the mean percentage of total brain fatty-acid concentration. Bold values highlight significant differences between two genotypes on a given diet

We analyzed the serum and brain tissue from animals maintained on the ω-3-deficient diet to confirm that this diet altered membrane lipid profiles (Tables 1 and 2). For both genotypes, the deficient diet led to significant reductions in the serum levels of ω-3 ALA, eicosapentaenoic acid (EPA), and DHA (*F*_(2,38)_ = 99.47-515.76, *p* < 0.001). The brain levels of DHA were also depleted by the deficient diet (NR1:6.29 ± 0.90, WT:6.92 ± 1.56) (*F*_(2,38)_ = 402.72–515.76, *p* < 0.001).Therefore, the ω-3-deficient diet reduced both brain and serum levels of ω-3 fatty acids as expected.

**Table 2:** Serum fatty-acid profile of WT and NR1KD mice on experimental diets

	Control diet		Omega-3 deficient		Omega-3 rich	
	WT	NR1	WT	NR1	WT	NR1
Omega-3 fatty acids						
α-linolenic acid (ALA)	0.439 ± 0.078	0.425 ± 0.090	0.031^**‡**^ ± 0.012	0.022^**‡**^ ± 0.011	0.057^**‡**^ ± 0.022	0.065^‡^ ± 0.010
Eicosapentaenoic acid (EPA)	0.193 ± 0.071	0.192 ± 0.061	0.016 ± 0.009	0.019 ± 0.005	2.443^**‡**^ ± 0.551	2.177^‡^ ± 0.547
Docosapentaenoic acid (DPA)	0.123 ± 0.029	0.117 ± 0.032	0.090 ± 0.036	**0.117** ^**†**^ ± 0.027	0.116 ± 0.026	0.093 ± 0.033
Docosahexaenoic acid (DHA)	4.999 ± 0.306	4.754 ± 0.395	0.981^**‡**^ ± 0.181	0.903^**‡**^ ± 0.108	8.006^**‡**^ ± 0.438	7.081^†‡^ ± 0.181
Omega-6 fatty acids						
Linoleic acid (LA)	28.952 ± 2.251	30.444 ± 3.657	29.744 ± 1.990	31.735 ± 1.652	29.563 ± 2.123	32.759^†^ ± 1.660
Eicosadienoic acid (EDA)	0.360 ± 0.055	0.373 ± 0.044	0.421 ± 0.083	0.488^**‡**^ ± 0.118	0.291 ± 0.051	0.335 ± 0.094
Dihomo-gamma-linolenic acid (DGLA)	1.037 ± 0.175	1.063 ± 0.166	0.841 ± 0.189	0.893 ± 0.256	1.327^**‡**^ ± 0.222	1.096 ± 0.138
Arachidonic acid (AA)	8.854 ± 1.482	8.785 ± 1.679	12.583^**‡**^ ± 0.981	10.737^**‡**^ ± 0.875	5.089^**‡**^ ± 0.511	4.420^†‡^ ± 0.572
Adrenic acid	0.162 ± 0.010	0.167 ± 0.030	0.464^**‡**^ ± 0.053	0.492^**‡**^ ± 0.039	0.063^**‡**^ ± 0.022	0.049^‡^ ± 0.018
Osbond acid	0.162 ± 0.028	0.249 ± 0.034	2.875^**‡**^ ± 0.334	**3.180** ^‡**†**^ ± 0.408	0.094 ± 0.025	0.075 ± 0.020
Omega-9 fatty acids						
Oleic acid	6.618 ± 0.733	6.617 ± 0.928	5.963 ± 0.469	5.652^**‡**^ ± 1.392	5.952 ± 0.590	5.193^†‡^ ± 0.495
Gondoic acid	0.393 ± 0.098	**0.251** ^**†**^ ± 0.059	0.321 ± 0.123	0.326 ± 0.145	0.221^**‡**^ ± 0.053	0.303 ± 0.108
Nervonic acid	0.285 ± 0.061	0.262 ± 0.049	0.290 ± 0.087	0.271 ± 0.059	0.244 ± 0.033	0.292 ± 0.071

Data includes males and females aged 10–15 weeks (control diet (*n*=): WT 4M, 4F; NR1 5M, 5F. Deficient diet (*n*=): WT 5M, 5F; NR1 5M, 5F. Supplemented diet (*n*=): WT 5M, 5F; NR1 5M, 4F). Omega-3-deficient diet reduced levels of DHA and increased omega-6 fatty-acid concentrations in both genotypes. Omega-3 rich increased EPA and DHA levels, while normalizing or reducing omega-6 fatty-acid levels. †*p* < 0.05 post-hoc comparison to wild-type on the same diet, ‡*p* < 0.05 post-hoc comparison to control diet within the same genotype. Data expressed as the mean percentage (%) of total brain fatty-acid concentration. Bold values highlight significant differences between two genotypes on a given diet

Further, in both genotypes the deficient diet significantly increased brain and serum levels of several ω-6 fatty acids. In fact, the levels of osbond acid in the brain were more than 20 times higher than what was detected for the control diet, and this was seen for both genotypes (NR1:7.84 ± 0.67, WT:7.00 ± 1.32) *F*_(2,44)_ = 342.02–452.39, *p* < 0.001). This is not surprising, since elevated osbond acid levels are used as an indicator of DHA deprivation.^19^ The ω-3-deficient diet also led to increased serum and brain levels of ω-6 fatty-acid AA (arachidonic acid), an initiator of inflammation (brain: *F*_(2,44)_ = 34.17–53.17, *p* < 0.001) (serum: *F*_(2,38)_ = 70.57–93.03, *p* < 0.001). Another ω-6 fatty acid, adrenic acid, was also elevated in both serum (*F*_(2,38)_ = 314.54–384.01, *p* < 0.001) and brain (*F*_(2,44)_ = 177.62–177.76, *p* < 0.001). There was also an effect of the ω-3-deficient diet on the serum levels of ω-9 oleic acid (*F*_(2,38)_ = 6.36, *p* < 0.05).

As expected, supplementation of dietary ω-3 changed brain and serum profiles in the opposite direction. For both genotypes, serum (*F*_(2,38)_ = 99.47–121.50, *p* < 0.001) and brain (*F*_(2,44)_ = 10.45–21.01, *p* < 0.001) levels of ω-3 EPA increased. The most substantial effect of supplementation was on the brain levels of ω-6 PUFAs, which were reduced. Brain levels of the ω-6 osbond acid, which were dramatically elevated by the deficient diet, were reduced by the supplement diet (NR1:0.15 ± 0.01, WT:0.16 ± 0.03) (*p* = 1). Arachidonic acid levels were also reduced by ω-3 supplementation, in both brain (*F*_(2,44)_ = 34.17–53.17, *p* < 0.001) and serum (*F*_(2,38)_ = 70.57–93.03, *p* < 0.001). In addition, brain levels of adrenic acid were reduced (NR1:2.25 ± 0.05, WT:2.30 ± 0.12) (*F*_(2,44)_ = 177.62–177.76, *p* < 0.001). The overall effect of supplementation was a reduction of ω-6 PUFAs in the brain and serum, while ω-3 PUFA levels were more notably changed in the serum.

Although the ω-3 supplement diet reduced the brain levels of ω-6 PUFAs, these still remained higher in NR1KD brains compared to WT. In fact, across all three diets, the NR1KD brain levels of ω-6 linoleic acid were higher than WT levels on the same diet (effect of genotype: *F*_(1,44)_ = 26.46, *p* < 0.05). Therefore, there was a main effect of genotype on ω-6 levels that was independent of diet.

Diet induced alterations in brain and serum PUFA levels were not always correlated (Tables 1 and 2). Across diets, brain levels of LA were consistently higher in NR1KD mice (*F*_(1,44)_ = 10.56–17.73, *p* < 0.01). Although serum levels did not show increased LA levels within each diet, the combined effect of diets were significant in NR1KD mice (*F*_(1,38)_ = 9.77, *p* < 0.01). The omega-3-rich diet reduced adrenic acid in the brain of mutant mice, while serum levels were unchanged. Thus, NMDA receptor deficiency differentially affects brain and serum lipid composition.

### Omega-3 deprivation selectively affects the survival of male mutant mice

**Fig. 1:**
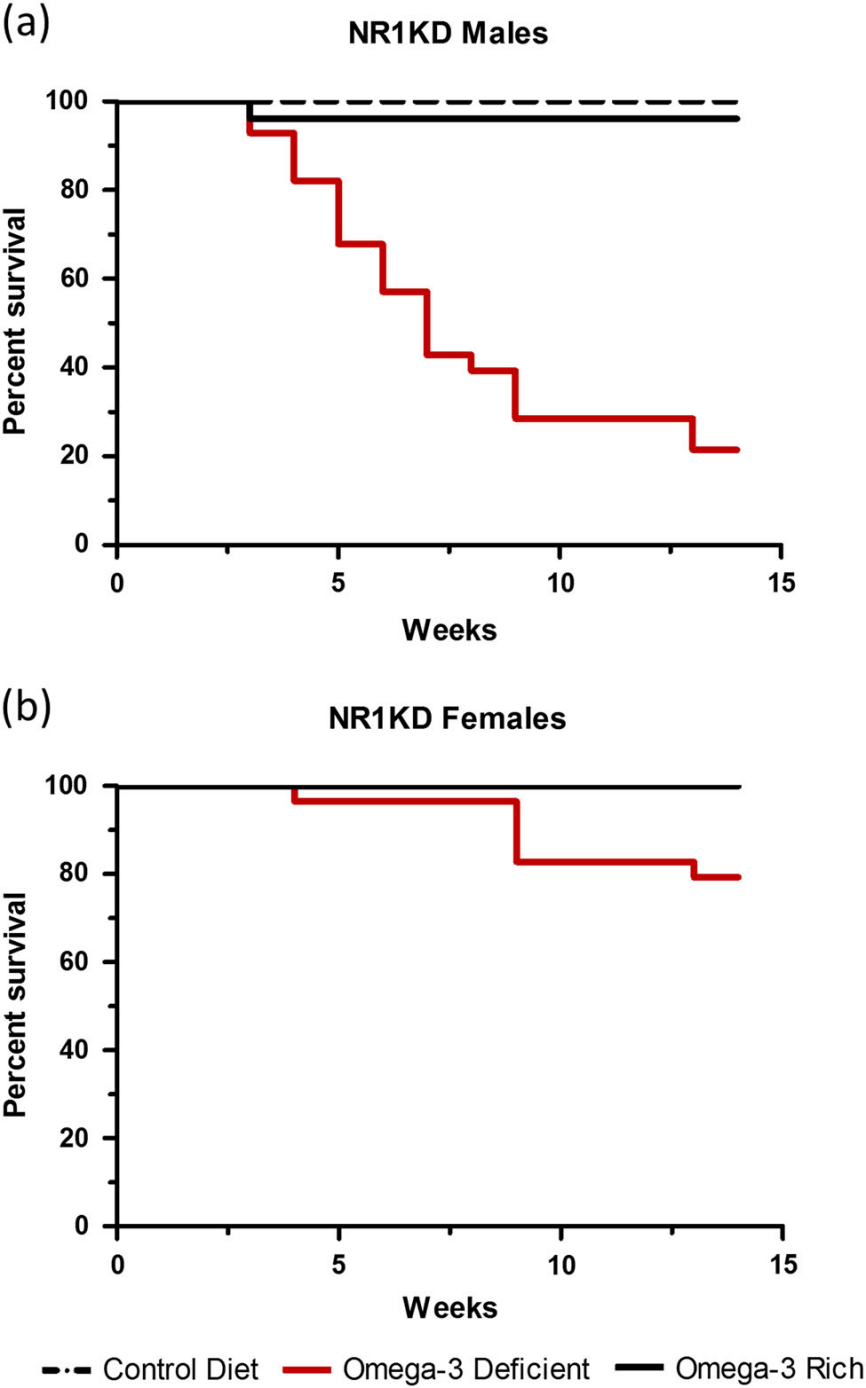
Kaplan–Meier survival curve for male and female NR1KD mice in three diets. **a** Survival curve for NR1KD males on control, omega-3-rich and deficient diets, aged 3–15 weeks. At 15 weeks, 21% of male NR1KD mice survived until the end of the study on the omega-3-deficient diet (*X* ^(2)^ = 49.01; *p* < 0.0001, *n* = 19–25 mice per group). Control diet and omega-3-rich diets did not affect survival of NR1KD mice (*p* = 0.39). **b** Survival curve for NR1KD females on control, omega-3-rich and deficient diets, aged 3–15 weeks. All females on the control diet and omega-3-rich diets survived until testing end point (*p* = 1.0). By the end of the study, 80% of female NR1KD mice survived on the omega-3-deficient diet (*X* ^(2)^ = 10.70; *p* < 0.005, *n* = 23–25 mice per group).

NR1KD males were particularly vulnerable to the dietary lack of ω-3. After observing high mortality rates among mutant males, Kaplan–Meier survival curves were generated for both genotypes on the three diets (Fig. 1). Survival of WT mice was not affected by diet. However, death of male NR1KD mice occurred steadily from the time of weaning. Only 21% of NR1KD male mice placed on the ω-3-deficient diet survived to 12 weeks of age, when behavioral testing began (*X* ^(2)^ = 49.01, *p* < 0.0001, *n* = 19–25) (Fig. 1a). The cause of death could not be determined, and there were no signs of weight loss or sickness behavior preceding death. Surviving ω-3-depleted males had similar body weights to mutant males that were maintained on the control and ω-3 enriched diets (Fig. S1).

In contrast, the majority of NR1KD female mice (80%) survived to testing age when maintained on the ω-3-deficient diet (*X* ^(2)^ = 10.70, *p* < 0.005, *n* = 23–25) (Fig. 1b). The body weight of female mutant mice was also not affected by diet (Fig. S1). Thus, while ω-3 deprivation reduced the survival of both male and female mutant mice, the effect was dramatically pronounced in males. There was no effect on survival for mutant mice that were maintained on the control diet or the ω-3 enriched diet (male *p* = 0.39; female *p* = 1) (Fig. 1).

### Dietary manipulations of omega-3 levels have minimal effects on locomotor activity, stereotypy, sociability, and sensorimotor gating

**Fig. 2:**
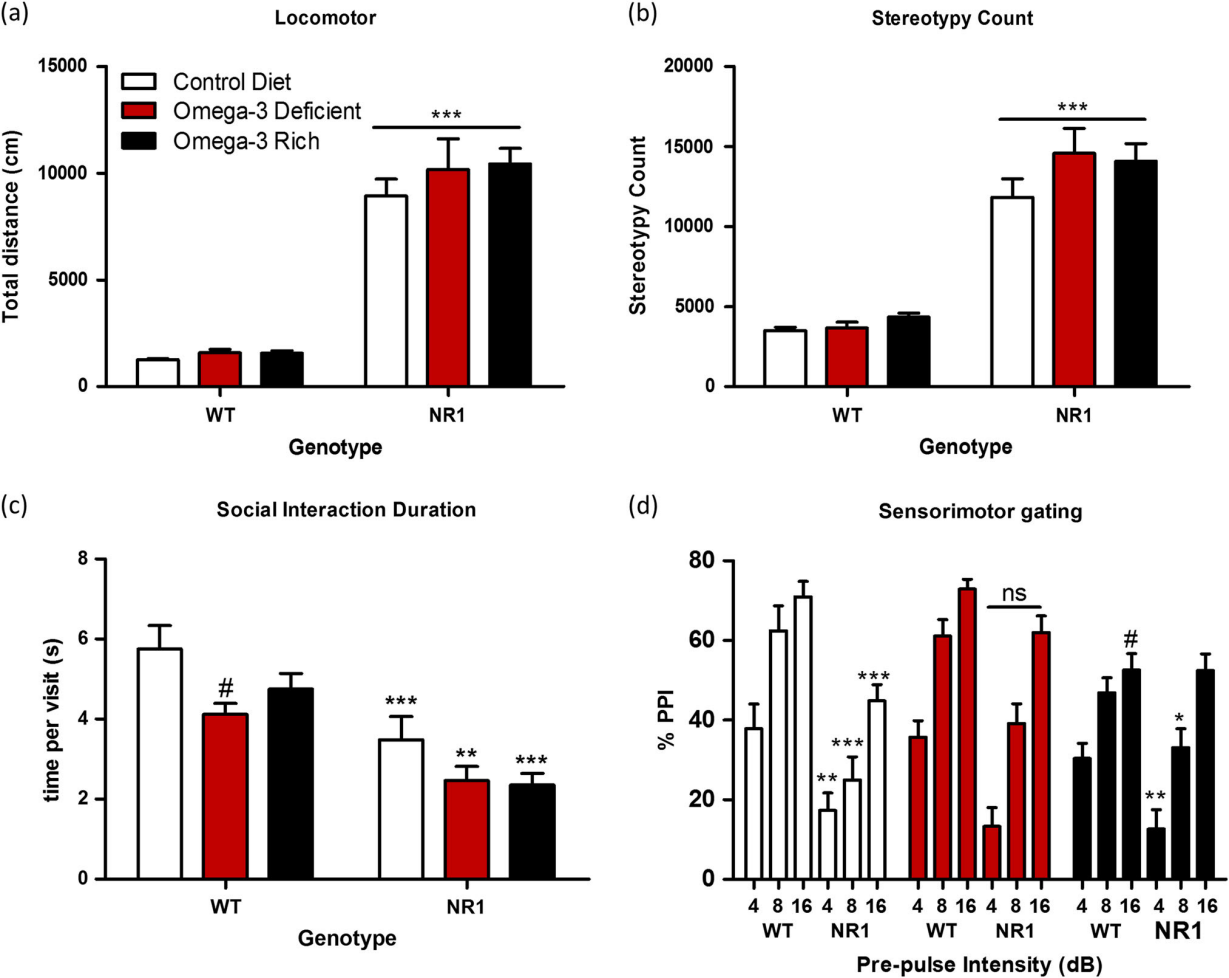
Minimal effect of diet on locomotor activity, sociability, and impaired sensorimotor gating of acoustic startle response in NR1-KD mice. Male and female mice aged 12–15 weeks were tested for locomotor activity, sociability, and pre-pulse inhibition of acoustic startle response using three prepulse intensities (4, 8, and 16 dB). **a** Locomotor activity in a novel environment was increased in NR1KD mice (*p* < 0.001), with no effect of diet on either genotype. Control diet (*n*=): WT 16M, 25F; NR1 10M, 21F. Deficient diet (*n*=): WT 14M, 15F; NR1 10M, 14F. Supplemented diet (*n*=): WT 13M, 17F; NR1 18M, 16F. **b** Repetitive behavior in a novel environment was increased in NR1KD mice with no effect of diet on either genotype (*p* < 0.001). **c** Social time per visit was longer in WT compared to NR1KD mice on all diets (*p* < 0.01–0.001). WT mice on the omega-3-deficient diet had reduced social time per visit compared to control diet (*p* < 0.001) (diet-genotype interaction: *p* < 0.05) Control diet (*n*=): WT 8M, 15F; NR1 13M, 7F. Deficient diet (*n*=): WT 14M, 14F; NR1 5M, 13F. Supplemented diet (*n*=): WT 13M, 19F; NR1 17M, 15F. **d** Sensorimotor gating (PPI) was impaired in NR1KD mice at 4 dB and 8 dB on omega-3 rich (*p* ≤ 0.02) and control diets (*p* ≤ 0.01), and at 16 dB on the control diet (*p* ≤ 0.001). No significant differences were found between WT and NR1KD mice on the omega-3-deficient diet (*p* = 0.06). At pre-pulse 16 dB, WT mice on omega-3-rich diet have reduced PPI compared to WT mice on omega-3-deficient (*p* ≤ 0.001) and control diet (*p* < 0.05). Control diet (*n*=): WT 7M, 10F; NR1 6M, 8F. Deficient diet (*n*=): WT 12M, 11F; NR1 8M, 12F. Supplemented diet (*n*=): WT 13M, 17F; NR1 18M, 18F. All statistics performed using multivariate two-way ANOVA, Bonferroni post-hoc. *p < 0.05 for within diet, across genotype comparison. #*p* < 0.05 for within genotype, across diet comparison. Data shown as mean ± SEM.

NR1KD mice showed increased locomotor activity relative to WT mice on all three diets (Fig. 2) (main effect of genotype *F*_(1172)_ = 296.81, *p* < 0.001). Similarly, NR1KD mice showed increased stereotypy across all diets (main effect of genotype *F*_(1170)_ = 179.44, *p* < 0.001). There was not a main effect of diet for WT (*F*_(2172)_ = 0.84, *p* = 0.92) and NR1KD mice (*F*_(2172)_ = 2.80, *p* = 0.063) (Fig. 2a). There was no effect of sex on locomotor activity and stereotypy across genotype or diet (*F*_(2170)_ = 2.59, *p* = 0.078) (Fig. S2). Strikingly, there was no beneficial effect of omega-3 supplementation on NR1KD mice (Fig. 2a).

We measured the effect of diet on social behavior by recording the amount of time that mice spent in a zone of close proximity to a novel, wild-type mouse of the same sex and age. We studied the quality of social interactions by measuring the average time that mice spent with each visit to the social zone. On the control diet, NR1KD mice spent significantly less time per social visit (*F*_(1135)_ = 7.59, *p* < 0.001) (Fig. 2c). There was no effect of ω-3 deficiency (*F*_(2136)_ = 6.44, *p* = 0.12) or supplementation (*F*_(2136)_ = 6.44, *p* = 0.84) on the sociability of NR1KD mice. However, in WT mice, ω-3 deprivation decreased the quality of social interaction, with mice spending less time per visit (*F*_(2135)_ = 5.67, *p* < 0.01) (Fig. 2c). Although there were sex-effects on the survival of NR1KD mice, there were no sex-effects on social interaction (Fig. S2).

Sensorimotor gating was evaluated by measuring the pre-pulse inhibition of acoustic startle response. On the control diet, NR1KD mice showed disruptions in sensorimotor gating, reflected by an inability to reduce startle response (4 dB pre-pulse, *F*_(1119)_ = 6.81, *p* = 0.01, 8 dB pre-pulse, *F*_(1119)_ = 16.52, *p* < 0.001, 16 dB pre-pulse, *F*_(1119)_ = 11.09, *p* = 0.001) (Fig. 2d). There was no effect of ω-3 deprivation or supplementation on NR1KD sensorimotor gating (Fig. 2d and Supplementary Fig. S3). Interestingly, WT mice showed a slight reduction in sensorimotor gating with the ω-3 supplement diet (16 dB:*F*_(2119)_ = 8.02, *p* < 0.05) and had a reduced baseline startle response (Fig. S4).

### Omega-3 deficiency exacerbates executive function impairments of NR1KD mice

**Fig. 3:**
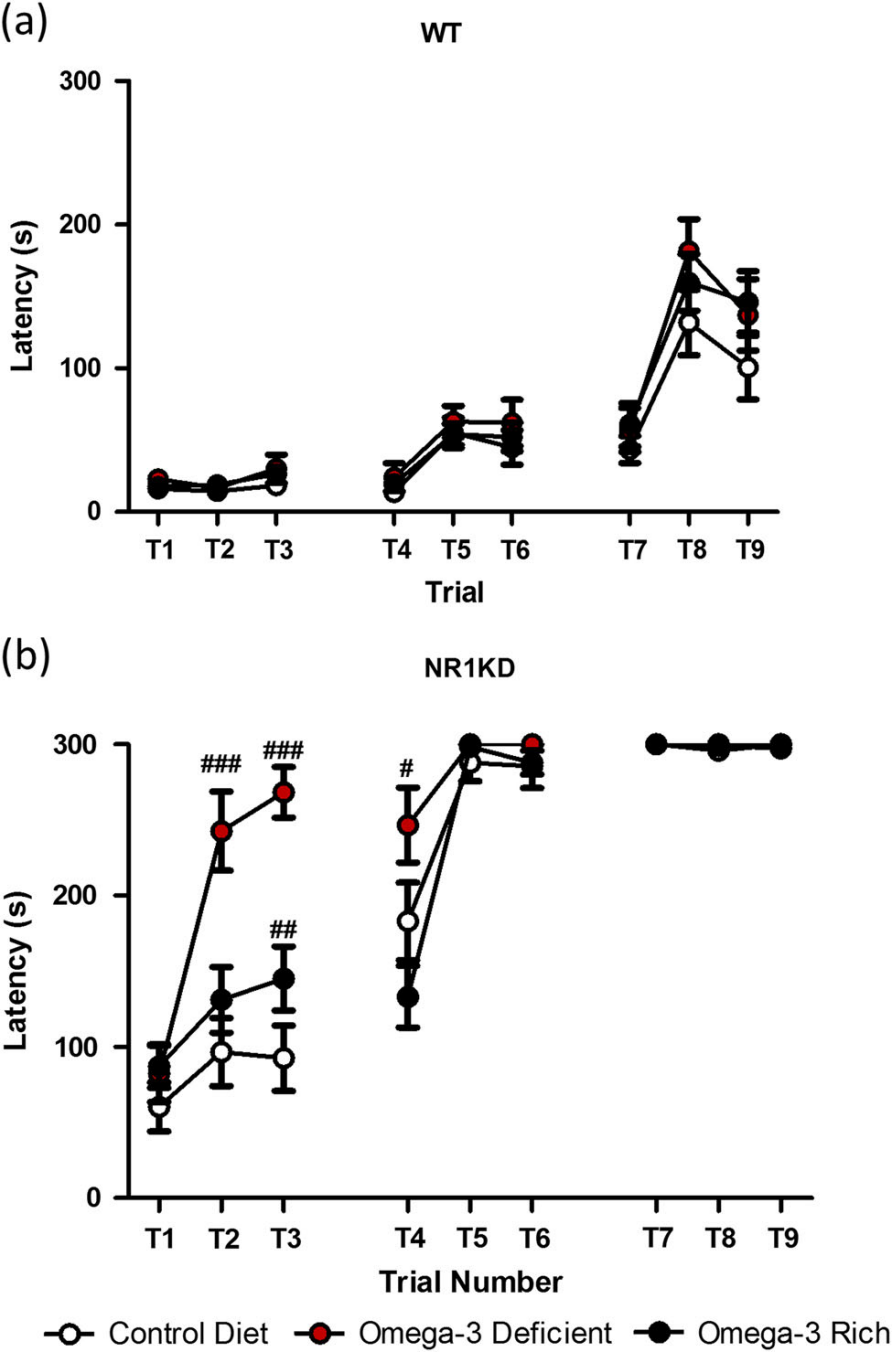
Omega-3-deficient diet worsens executive function in NR1KD mice. Adult male and female mice aged 12–15 weeks were tested over 3 days for executive function using a goal-oriented puzzle box task. Executive function was measured by the latency to reach a goal box, with a 5 min time limit. Genotype differences were found in each trial between WT and NR1KD mice in the three diets (*p* < 0.05). **a** No significant difference was observed in WT mice on the different diets. **b** NR1KD mice on the omega-3-deficient diet required more time to perform tasks on trials T2-T4 compared to the control diet (*p* < 0.05–0.001). Diet-genotype interaction at T2-T4: *p* ≤ 0.001. All statistics performed using repeated measures ANOVA, Bonferroni post-hoc. Data shown as mean ± SEM. ^#^ *p* < 0.05, ^##^ *p* < 0.01, ^###^ *p* < 0.001 for within genotype, across diet comparison. Control diet (*n*=): WT 8M, 15F; NR1 6M, 7F. Deficient diet (*n*=): WT 15M, 15F; NR1 7M, 13F. Supplemented diet (*n*=): WT 13M, 19F; NR1 13M, 15F.

Executive function and problem solving were assessed using the puzzle box behavioral paradigm. This test measures the amount of time required for a mouse to reach a goal box with increasingly challenging obstacles. On the control diet, NR1KD mice had impaired executive function compared to WT (over Trials T1-T9:*F*_(1134)_ = 5.03–178.40, *p* < 0.05) (Fig. 3). Omega-3 deprivation worsened the performance of NR1KD mice on trials T2-T4, in which the mouse must use an underpass to reach the goal box (T2:*F*_(2134)_ = 17.30, *p* < 0.001; T3:*F*_(2134)_ = 25.05, *p* < 0.001; T4:*F*_(2134)_ = 13.16, *p* < 0.05) (Fig. 3b). Male mice on the ω-3-deficient diet had a greater impairment in executive function compared to female mice (Fig. S5). Although ω-3 deprivation worsened the executive function of NR1KD mice, ω-3 supplementation did not improve their performance (Trials T1-T9:*F*_(2134)_ = 0.012–25.05, *p* < 0.01–1) (Fig. 3b). Notably, there was no effect of diet on the performance of WT animals in this test.

## Discussion

Erythrocyte and serum fatty-acid measurements are the most accessible indicators for the status of PUFAs in living patients. There is evidence in the literature that blood and serum levels of PUFAs reflect brain PUFA composition.^20^ Indeed, we also found that dietary ω-3 depletion lowered EPA and DHA in both brain and serum, and elevated the levels of several ω-6 PUFAs. Manipulation of diet was well correlated between brain and serum levels of ω-3, ω-6, and ω-9 fatty acids. However, the effects of diet on omega-3 levels were more robustly observed in serum than in the brain. While supplementation increased DHA levels in the serum, there was no such increase in the brain.

On the other hand, our studies also revealed that, when NMDAR deficiency occurs, there are changes in brain fatty acids that are not reflected in the serum. Across diets, there were consistently elevated levels of LA in the NR1KD brain, but there was no such change in serum. Therefore, there are likely brain-specific changes that occur as a consequence of NMDAR hypofunction, and caution should be used when interpreting patient serum results to infer the status of brain fatty-acid profiles.

Our studies in mice were performed to ask whether impairments in NMDA receptor signaling heighten the neurological vulnerability to ω-3 deficiency. We found that NMDA receptor hypofunction has a basal effect of altering brain lipid composition, generally increasing the levels of ω-6 LA. To our knowledge, this is the first demonstration that genetic NMDA receptor hypofunction affects brain or serum PUFA composition. NMDA receptor mediated Ca2 + activation of phospholipase 2A can cause a release of AA from phospholipids.^21^ It is conceivable that impaired NMDA receptor signaling would reduce the production of AA, however, we did not see any significant difference in brain AA concentrations when mutant mice were maintained on the control diet. Instead, we found increased levels of LA in NR1KD mice on the control diet. The observed increase in LA might reflect a compensatory mechanism to increase NMDA receptor signaling in NR1KD mice, since LA reduces the Mg2 + block of NMDA receptors.^22^ Therefore, alterations in PUFA composition within NR1KD mice might act to potentiate NMDA receptor signaling and compensate for deficiency in this key neurotransmitter system.

The diet manipulations impacted both wild-type and mutant mice in the same direction, where ω-3 supplementation increased the ω-3:ω-6 ratio in the brain, and ω-3 deficiency decreased the ratio. Changes in the ratio of ω-3 to ω-6 are likely to have biological effects, particularly on inflammatory processes.^23^ Both ω-3 and ω-6 fatty acids are metabolized into lipid signaling molecules by the same enzymes, but the ω-6 fatty acids produce pro-inflammatory prostanoids, whereas the ω-3 fatty acids produce anti-inflammatory and pro-resolving molecules.^24^ Since a consistent outcome of NMDAR deficiency was a decrease in the ratio of ω-3:ω-6 in the brain, it is likely that this promotes pro-inflammatory signaling in NR1KD mice. Altered ω-3:ω-6 ratios can also lead to differences in membrane fluidity, intracellular signaling and can affect neurotransmitter signaling.^25^ It has been shown that increased ω-6 levels modulate adenylyl cyclase and protein kinase A activity, and can thus potentiate serotonergic and catecholamingeric signaling through increases in their downstream kinase activity.^25^

The cumulative effects of a ω-3-deficient diet led most drastically to increased mortality in NR1KD mice. Male mice were particularly vulnerable and suffered surprisingly high rates of mortality. This was unexpected, since ω-3 deficiency does not increase mortality in wild-type adult mice or rats, even after generations of depletion.^26, 27^ In our studies, we were unable to determine the cause of death for these animals and there were no indications of illness prior to their death. We investigated whether male NR1KD had a different lipid profile than female NR1KD mice on any of the three diets. Neither brain nor serum results pointed towards a sex-dependent effect of omega-3 deficiency on fatty-acid levels (Tables S2–S3). The exception may be increases in serum levels of eicosadienoic acid in NR1KD males. However, lipid profiles were obtained from males that survived to at least 10 weeks of age, which could bias the data to exclude those males that die before 10 weeks.

Since NR1KD mice are vulnerable to seizures on the control diet,^28^ we hypothesize that sudden death from seizure occurred. This hypothesis stems from our previous studies, which showed that DHA supplementation reduces seizure susceptibility in chemical and kindling models of epilepsy.^29–32^ Some (but not all) clinical studies report that ω-3 supplements reduce seizure frequency in patients with epilepsy.^33, 34^ Since ω-3 supplements are associated with reduced seizure severity, we hypothesize that dietary depletion increases seizure frequency or intensity in NR1KD mice. In females, estrogen can also be protective against seizure-induced neuron loss,^35^ which could explain why NR1KD female mice had a lower mortality rate than males on the omega-3-deficient diet.

Despite the high rates of mortality among NR1KD mice, ω-3 deprivation had comparatively modest effects on most of the behaviors that we assessed. A previous study in rats reported hyperlocomotion after two generations of ω-3 depletion; however, we observed no diet effect on locomotor activity and stereotypy.^35^ It should be noted that the previous study also reported that there were not locomotor abnormalities in first generation ω-3-deficient mice.^35^ Social interactions were affected by genotype, but not by diet. The exception to this general conclusion was the decrease in social interaction that was observed for wild-type mice maintained on the deficient diet.

We assessed problem solving and executive function by presenting the mouse with new obstacles to overcome in order to reach a goal box. NR1KD mice on the control diet were able to overcome the simplest obstacle and use an underpass to reach the goal. However, ω-3 deprivation further impaired their performance in this test, and mutant mice were unable to solve the simplest task of using an underpass to reach the goal. An ω-3 supplemented diet did not have a beneficial effect, and in fact mutant performance was further impaired by ω-3 supplementation in one trial of the test.

We did not observe a worsening in sensorimotor gating, despite other reports that ω-3 deprivation can impair sensorimotor gating in wild-type mice.^36, 37^ Discrepancies between previous findings and our study can be partially attributed to differences in the age of mice used to study acoustic startle response. While previous studies have used eight-week-old or younger mice, we used mice aged 11–15 weeks for our behaviors. Sensorimotor gating can be affected by the testing age, where deficits are more pronounced in younger mice.^38, 39^ In addition, genetic background can affect sensorimotor gating.^40^ Our study used mice on a genetically-defined, F1 C57/129 background, and not the C57Bl/6J strain used in Federova *et al*.^37, 38^ It is interesting that the ω-3 supplemented diet produced impairments in the wild-type acoustic startle response. A possible explanation for this phenomenon is the effect of diet on ω-6 levels in the brain. We consistently observed that ω-3 supplementation led to decreases in ω-6 levels, which can reduce downstream serotonergic signaling that is required in sensorimotor gating.^25, 41^ We also found that ω-3 deficiency did not further perturb acoustic startle response in NR1KD mice. This may reflect a basement effect among NR1KD mice, particularly at lower decibel prepulse intensities where no PPI is observed.

Indeed there were no tests in which ω-3 supplementation improved the behavioral abnormalities of NR1KD mice. The supplement diet increased the levels of ω-6 LA, and reduced the levels of ω-6 AA and adrenic acid in brain and serum of both genotypes. Interestingly, reduction in AA changes membrane fluidity, which can further impair some behaviors.^25, 42^ Supplementation also increased ω-3 EPA, but did not increase DHA, the most abundant ω-3 PUFA in the brain. Despite these changes in lipid composition, the supplement diet did not improve any of the behavioral abnormalities of NR1KD mice. These results suggest that a balanced ratio of ω-3:ω-6 may be required in maintaining an optimal level of lipid signaling within the brain.^43^ If the ratio imbalance in NR1KD mice is due to higher levels of ω-6 LA, then manipulation of dietary LA could improve brain function where dietary EPA and DHA could not.

We describe these findings in the context of schizophrenia because NMDAR hypofunction is implicated in the disorder.^12, 44^ Several studies have shown that patients with schizophrenia experience some improvements in symptoms with dietary ω-3 supplementation.^3, 4, 8–10^ However, there are also clinical studies where ω-3 supplementation did not affect symptom outcomes.^45, 46^ Since supranormal levels of ω-3 did not improve any of the behavioral phenotypes of NR1KD mice, we hypothesize that the beneficial effects of supplements in humans may be due to a restoration of ω-3 levels in individuals deficient for ω-3 PUFAs.^47^ This may also explain why some patients, who do not have a baseline ω-3 deficiency, do not benefit from supplementation.

Importantly, our studies show that alterations in NMDA receptor signaling change PUFAs in the brain in ways that heighten the vulnerability to dietary deficits. We propose that the beneficial effects of ω-3 supplementation are likely the result of correcting abnormal ω-3:ω-6 ratios. This supplementation may be particularly critical for male patients who have a genetic vulnerability to low levels of dietary ω-3 fatty acids. Behavioral deficits from NMDA receptor knockdown were not improved with omega-3 supplementation, demonstrating that supranormal levels of DHA cannot overcome impairments to this critical neurotransmitter system. Further studies using omega-3 supplementation among other mouse models of psychiatric disorders may elucidate subgroups of individuals that could benefit from omega-3 supplementation.

## Methods

### Animals

All procedures were conducted in accordance with the University of Toronto Faculty of Medicine and Pharmacy Animal Care Committee and with CCAC guidelines for the care and use of animals. NR1KD mice, with a targeted hypomorphic mutation of *Grin1* (*GluN1*), were generated as described.^11^ Congenic C57Bl/6J*Grin1* + /− and 129 × 1/SvJ*Grin1* + /− mice were crossed to produce experimental F1 mice as recommended by the Banbury Conference.^48^

Behavioral tests were performed on male and female WT and NR1KD mice ages 12–15 weeks. Mice were housed 1–4 per cage with a 12 h light–dark cycle (7:00 a.m.–7:00 p.m.) with ad libitum access to food and water. Mice were tested during the light cycle (9:00 a.m.–5:00 p.m.) without access to food or water for the duration of the test. Mice were naive to all tests performed. Twenty-four hours after the last behavioral test, mice were killed for brain and serum fatty-acid analysis. Experimenters were not blind to diet or genotype.

### Diet

Breeding pairs and weaned pups were maintained on one of three diets as previously described^49^ (Table S1): 1. Regular diet (Teklad 2018 chow, 10% fat with ratio of ω-6 to ω-3 = 10:1) 2. Omega-3-deficient diet (10% fat supplied from safflower oil with ratio of ω-6 to ω-3 = 760:1) 3. Omega-3 enriched diet (10% fat from: menhaden oil (2%) and safflower oil (8%), with ratio of ω-6 to ω-3 = 4:1). These diets all had the same fat content and caloric value.

### Locomotor activity and stereotypy

Mice were placed in a Plexiglass chamber (20 × 20 × 45 cm^3^) for 2 h. Total distance travelled and stereotypy number were measured in 5 min bins by infrared beam breaks using Versamax activity monitors (Omnitech Electronics, Columbus, OH, USA). Stereotypy number refers to sessions of repetitive grooming behavior, which was automatically scored using the Versamax activity monitor. Control diet: *n* = WT 16M, 25F; NR1 10M, 21F. Deficient diet: *n* = WT 14M, 15F; NR1 10M, 14F. Supplemented diet: *n* = WT 13M, 17F; NR1 18M, 16F.

### Social interaction

Social behavior was assessed with a modified ”three-chamber test” as previously described.^13^ Mice were placed in an open arena with two wire cages. One cage was empty, and the other contained a novel sex- and age-matched wild-type mouse of the same genetic background as the test subject. Mice were allowed to freely explore both cages in the arena for 10 min and were video tracked using Viewer2 software (Biobserve, St Augustin, Germany). Social visit duration was measured as time spent in the 5 cm zone around the novel mouse divided by the number of visits. Control diet: *n* = WT 8M, 15F; NR1 13M, 7F. Deficient diet: *n* = WT 14M, 14F; NR1 5M, 13F. Supplemented diet: *n* = WT 13M, 19F; NR1 17M, 15F.

### Pre-pulse inhibition of acoustic startle response

Pre-pulse inhibition of the acoustic startle response was measured with SR-LAB equipment and software from San Diego Instruments. Accelerometers were calibrated to 700 ± 5 mV and output voltages were amplified and analyzed for voltage changes using SR Analysis (San Diego Instruments, San Diego, CA, USA), exported as an excel file and statistically analyzed on SPSS v19 (IBM, Armonk, NY, USA). Background white noise was maintained at 65 dB. PPI was measured in a 30 min test with 80 randomized trials of: pulse alone (100 dB above background), pre-pulse alone (4, 8, or 16d B above background), pre-pulse plus pulse and no pulse. Five pulse alone trials were performed at the start and end of the 80 trials. The onset of the pulse following the pre-pulse was delayed 100 ms. Time interval between trials were randomized from 5s-20s. Pre-pulse inhibition was measured as a decrease in the amplitude of startle response to a 100 dB acoustic startle pulse, following each pre-pulse (4, 8, and 16 dB). Control diet: *n* = WT 7M, 10F; NR1 6M, 8F. Deficient diet: *n* = WT 12M, 11F; NR1 8M, 12F. Supplemented diet: *n* = WT 13M, 17F; NR1 18M, 18F.

### Puzzle box test

The puzzle box test was performed as previously described.^13^ Mice were tested on three trials over three consecutive days for a total of nine trials (T1-T9). The puzzle box was a brightly lit arena (58 × 28 × 27.5 cm^3^) connected to a darkened goal box (14 × 28 × 27.5 cm^3^) by a wall divider. In trial T1 mice used a doorway and underpass to reach the goal box. In trials T2, T3, and T4 the doorway was blocked leaving an open underpass. In trials T5, T6, and T7 the underpass was blocked with corncob bedding. In trials T8 and T9 the underpass was blocked with a removable plug. Executive function was measured by the latency to reach the goal chamber, with a 5-min cutoff. Mice that failed to reach the goal on T1 were excluded from further testing. Control diet: *n* = WT 8M, 15F; NR1 6M, 7F. Deficient diet: *n* = WT 15M, 15F; NR1 7M, 13F. Supplemented diet: *n* = WT 13M, 19F; NR1 13M, 15F.

### Brain and serum collection

Samples were obtained from mice at 10–16 weeks of age (Control diet: *n* = WT 4M, 4F; NR1 5M, 5F. Deficient diet: *n* = WT 5M, 5F; NR1 5M, 5F. Supplemented diet: *n* = WT 5M, 5F; NR1 5M, 4F). Blood was collected from intracardial punctures performed on avertin-anesthetized mice (250 mg/kg), and brains were rapidly dissected and frozen in chilled isopentane before storage at −80 °C. Blood samples were left at room temperature for 30 min, prior to centrifugation at 1000×*g* at 4 °C. Supernatant was collected and stored at −80 °C until lipid analysis was performed.

### Lipid extraction

Total lipids were extracted using the Folch method.^50^ Prior to extraction, brains were weighed and serum volume was measured. Total lipids were extracted in chloroform:methanol:0.88% KCl in a ratio of 4:2:1.75, and a glass homogenizer was used to homogenize the brain. In all, 946 mg and 10 µg of heptadecanoic acid (Nu-Chek-Prep, Elysian, MN, USA) was added prior to extraction for brain and blood, respectively, as an internal standard. A second wash of 4 ml chloroform was performed. Total lipids were dried by N_2_ and reconstituted in 2 ml and 300 µl hexane for brain and serum, respectively.

### Methylation

From total lipid extract, 250 µl was added to 1.75 ml hexane and 2 ml 14% methanolic boron trifluoride (BF3 Sigma, Oakville, CA) and methylated for 1 h at 100 °C. For serum total lipids, 300 µl was added to 1 ml of 14% BF3 and methylated for 1 h at 100 °C. Samples were cooled for 10 min and 2 ml dH_2_O was added to the tubes and 1 ml of hexane added to the serum samples. Samples were centrifuge for 10 min and the hexane layer was extracted. Samples were dried down by N_2_ and reconstituted in 500 µl hexane.

### Fatty acid methyl ester analysis

Fatty acid methyl esters (FAME) were analyzed on a Varian-430 gas chromatograph (Varian, Lake Forest, CA) equipped with an Agilent capillary column (DB-23; 30 m × 0.25 mm i.d. × 0.25 µm film thickness, Santa Clara, CA). One microliter of FAME was injected in splitless mode. The carrier gas was helium, set to a constant flow rate of 0.7 ml/min. The injector and detector ports were set at 250 °C. FAME were eluted using a temperature program set initially at 50 °C for 2 min, followed by a ramp-up at 20 °C/min to 170 °C, and a hold at 170 °C for 1 min, and an increase of 3 °C/min to 212 °C and a hold at 212 °C for 5 min. Peaks were confirmed by identifying the retention times of authentic FAME standards of known composition (Nu-Chek-Prep, Elysian, MN, USA). Fatty-acid concentrations (nmol/g of brain) were calculated by proportional comparisons of the gas chromatography peak areas with that of the heptadecanoic acid internal standard.

### Statistical analysis

All statistical tests were performed using SPSS Statistics Software v19 (IBM, New York) and data were graphed using GraphPad Prism5 (GraphPad Software Inc., La Jolla, CA, USA). Statistical significance (*P* < 0.05) was determined using two-way univariate analysis of variance (ANOVA) and repeated measures ANOVA with Bonferroni post-hoc, as required for genotype, diet and sex comparisons. Kaplan–Meier survival plot was graphed and analyzed using GraphPad Prism5. Grubbs test (GraphPad Software Inc., La Jolla, CA, USA) was used to exclude outliers greater than two standard deviations from the mean. Behavioral measures were presented as mean ± SEM and fatty-acid measurements were presented as mean ± SD.

The sample size for behavioral measures were powered for detection of genotype and diet effects (1-beta ≥ 0.79), determined using SPSS Statistics Software v19 (IBM, New York). Sample size for fatty-acid analysis was powered for genotype, diet, and sex effects (1-beta ≥ 0.98).

## Electronic supplementary material

Supplementary Table 1

Supplementary Table 2

Supplementary Table 3

Supplementary Figure 1

Supplementary Figure 2

Supplementary Figure 3

Supplementary Figure 4

Supplementary Figure 5
