# Supplementary material for: Vulnerability to omega-3 deprivation in a mouse model of NMDA receptor hypofunction
Source: NPJ Schizophr. 2017 Mar 22;3:12. doi: 10.1038/s41537-017-0014-8 (PMC5441542; doi:10.1038/s41537-017-0014-8)
Supplement: Supplementary file 1 — Supplementary Table 1 [file 41537_2017_14_MOESM1_ESM.docx]

|  | **10% Safflower Oil** | **2% menhaden oil + 8% safflower oil** | **Standard chow** |
| --- | --- | --- | --- |
| **FA levels (% of total FA)** |  |  |  |
| **16:0** | 6.4 | 8.3 | 11.3 |
| **18:0** | 2.3 | 2.4 | 3.2 |
| **18:1n-9** | 12.0 | 6.5 | 19.4 |
| **18:2n-6** | 78.9 | 65.0 | 50 |
| **18:3n-3** | 0.1 | 0.4 | 4.8 |
| **20:5n-3** | 0 | 2.8 | - |
| **22:6n-3** | 0 | 2 | - |
