# Supplementary material for: Vulnerability to omega-3 deprivation in a mouse model of NMDA receptor hypofunction
Source: NPJ Schizophr. 2017 Mar 22;3:12. doi: 10.1038/s41537-017-0014-8 (PMC5441542; doi:10.1038/s41537-017-0014-8)
Supplement: Supplementary file 2 — Supplementary Table 2 [file 41537_2017_14_MOESM2_ESM.docx]

| **Omega-3 Fatty Acids** | | **Control diet** | | | | | | **Omega-3 Deficient** | | | | **Omega-3 Rich** | | | |
| --- | --- | --- | --- | --- | --- | --- | --- | --- | --- | --- | --- | --- | --- | --- | --- |
|  |  | WT | | | | NR1 | | WT | | NR1 | | WT | | NR1 | |
|  |  | F | | M | | F | M | F | M | F | M | F | M | F | M |
| **ALA** | 0.02±0.04 | | 0.04±0.03 | | 0.07±0.02 | | 0.06±0.01 | 0.04±0.03 | 0.05±0.01 | 0.03±0.03 | 0.04±0.03 | 0.04±0.03 | 0.06±0.02 | 0.02±0.03 | 0.04±0.03 |
| **EPA** | 0.06 ±0.02 | | 0.05±0.03 | | 0.04±0.02 | | 0.04 ±0.02 | 0.04 ±0.03 | 0.04 ±0.02 | 0.03 ±0.02 | 0.04 ±0.02 | 0.09 ±0.01 | 0.10 ±0.05 | 0.12 ±0.01 | 0.15 ±0.03 |
| **DPA** | 0.65 ±0.05 | | 0.64 ±0.15 | | 0.64 ±0.15 | | 0.66 ±0.12 | 0.54 ±0.00 | 0.67 ±0.12 | 0.58 ±0.13 | 0.69 ±0.12 | 0.60 ±0.09 | 0.67 ±0.06 | 0.67 ±0.06 | 0.63 ±0.16 |
| **DHA** | 14.06 ±0.14 | | 14.06 ±0.21 | | 14.10 ±0.58 | | 14.32 ±0.74 | 8.32 ±0.99 | 6.48 ±0.94 | 6.25 ±0.64 | 6.40 ±1.20 | 15.23 ±0.44 | 14.40 ±0.48 | 14.94 ±0.08 | 14.27 ±0.86 |
| **Omega-6 Fatty Acids** | | | | | | | | | | | | | | | |
| **LA** | 0.66 ±0.07 | | 0.68 ±0.12 | | 0.77 ±0.06 | | 0.80 ±0.03 | 0.85 ±0.02 | 0.80 ±0.02 | 0.99 ±0.09 | 0.96 ±0.02 | 0.81 ±0.02 | **0.94***±0.06 | 0.86 ±0.03 | **0.98***±0.03 |
| **EDA** | 0.30 ±0.03 | | 0.30 ±0.02 | | 0.35 ±0.07 | | 0.35 ±0.03 | 0.41 ±0.02 | 0.43 ±0.05 | 0.45 ±0.05 | 0.44 ±0.08 | 0.33 ±0.04 | **0.44*** ±0.15 | 0.35 ±0.05 | 0.38 ±0.06 |
| **DGLA** | 0.32 ±0.02 | | 0.37 ±0.03 | | 0.52 ±0.20 | | 0.45 ±0.12 | 0.34 ±0.08 | 0.54 ±0.24 | 0.45 ±0.32 | 0.40 ±0.14 | 0.66 ±0.21 | 0.63 ±0.14 | 0.58 ±0.09 | 0.73 ±0.11 |
| **AA** | 7.60 ±0.19 | | 7.31 ±0.13 | | 7.52 ±0.24 | | 7.53 ±0.30 | 8.14 ±0.30 | 8.43 ±0.42 | 8.17 ±0.15 | 8.28 ±0.35 | 6.96 ±0.19 | 6.59 ±0.13 | 7.01 ±0.37 | 6.96 ±0.27 |
| **Adrenic acid** | 2.81 ±0.03 | | 2.63 ±0.04 | | 2.82 ±0.21 | | 2.72 ±0.16 | 3.66 ±0.24 | 3.74 ±0.24 | 3.68 ±0.12 | 3.70 ±0.09 | 2.39 ±0.07 | 2.18 ±0.03 | **2.30*** ±0.01 | 2.21 ±0.04 |
| **Osbond acid** | 0.31 ±0.08 | | 0.30 ±0.01 | | 0.32 ±0.07 | | 0.32 ±0.03 | 6.09 ±1.47 | **7.54*** ±1.14 | 8.04 ±0.26 | 7.55 ±0.85 | 0.16 ±0.04 | 0.16 ±0.03 | 0.15 ±0.00 | 0.16 ±0.01 |
| **Omega-9 Fatty Acids** | | | | | | | | | | | | | | | |
| **Oleic acid** | 18.61 ±0.71 | | 18.80 ±1.29 | | 18.22 ±0.50 | | 17.71 ±0.13 | 16.93 ±0.53 | 16.63 ±0.47 | 16.69 ±0.62 | 17.11 ±1.35 | 18.96 ±0.33 | 18.73 ±0.33 | 18.05 ±0.48 | 19.04 ±0.60 |
| **Gondoic acid** | 2.36 ±0.05 | | 2.33 ±0.04 | | 2.19 ±0.12 | | 2.13 ±0.06 | 2.10 ±0.1 | 2.15±0.16 | 2.05 ±0.22 | 2.04 ±0.17 | 2.40 ±0.14 | 2.29 ±0.14 | 3.46 ±1.17 | **2.37*** ±0.19 |
| **Nervonic acid** | 1.13 ±0.02 | | 1.18 ±0.26 | | 1.07 ±0.22 | | 1.03 ±0.23 | 1.10 ±0.05 | 1.29 ±0.21 | 1.09 ±0.16 | 1.23 ±0.10 | 1.09 ±0.11 | 1.26 ±0.21 | 0.92 ±0.09 | 0.93 ±0.31 |

Abbreviations: ALA - α-linolenic acid, EPA - eicosapentaenoic acid, DPA - docosapentaenoic acid, DHA - docosahexaenoic acid, LA - linoleic acid, EDA - eicosadienoic acid, DGLA - dihomo-gamma-linolenic acid and AA - arachidonic acid.
