# Supplementary material for: Vulnerability to omega-3 deprivation in a mouse model of NMDA receptor hypofunction
Source: NPJ Schizophr. 2017 Mar 22;3:12. doi: 10.1038/s41537-017-0014-8 (PMC5441542; doi:10.1038/s41537-017-0014-8)
Supplement: Supplementary file 3 — Supplementary Table 3 [file 41537_2017_14_MOESM3_ESM.docx]

| **Omega-3 Fatty Acids** | **Control diet** | | | | | | | | **Omega-3 Deficient** | | | | | | | | **Omega-3 Rich** | | | | | | | |
| --- | --- | --- | --- | --- | --- | --- | --- | --- | --- | --- | --- | --- | --- | --- | --- | --- | --- | --- | --- | --- | --- | --- | --- | --- |
|  | WT | | | | NR1 | | | | WT | | | | NR1 | | | | WT | | | | NR1 | | | |
|  | F | | M | | F | | M | | F | | M | | F | | M | | F | | M | | F | | M | |
| **ALA** | 0.47 ±0.09 | | 0.41 ±0.07 | | 0.42 ±0.13 | | 0.43 ±0.03 | | 0.03 ±0.01 | | 0.03 ±0.09 | | 0.02 ±0.01 | | 0.02 ±0.00 | | 0.07 ±0.02 | | 0.05 ±0.02 | | 0.07 ±0.01 | | 0.06 ±0.01 | |
| **EPA** | 0.25 ±0.03 | | 0.14  ±0.04 | | 0.24 ±0.02 | | 0.14 ±0.04 | | 0.02 ±0.01 | | 0.01 ±0.01 | | 0.02 ±0.01 | | 0.02 ±0.00 | | 2.23 ±0.31 | | 2.37 ±0.54 | | 1.91 ±0.25 | | 2.14 ±0.33 | |
| **DPA** | 0.13 ±0.02 | | 0.11 ±0.03 | | 0.11 ±0.04 | | 0.13 ±0.01 | | 0.11 ±0.03 | | 0.07 ±0.03 | | 0.11 ±0.03 | | 0.12 ±0.03 | | 0.11 ±0.01 | | 0.12 ±0.04 | | 0.07 ±0.03 | | 0.12 ±0.02 | |
| **DHA** | 5.01 ±0.24 | | 4.99 ±0.52 | | 4.97 ±0.46 | | 4.54 ±0.19 | | 1.11 ±0.10 | | 0.81 ±0.07 | | 0.95 ±0.11 | | 0.84 ±0.07 | | 7.86 ±0.71 | | 8.10 ±0.28 | | 6.84 ±0.91 | | 7.42 ±0.43 | |
| **Omega-6 Fatty Acids** | | | | | | | | | | | | | | | | | | | | | | | | |
| **LA** | 27.01 ±0.68 | **30.9*** ±0.96 | | 27.57 ±3.03 | | **33.3*** ±1.22 | | 30.01 ±1.63 | | 29.39 ±2.75 | | 31.86 ±2.01 | | 31.57 ±1.43 | | 30.29 ±1.20 | | 29.79 ±2.40 | | 32.24 ±2.12 | | 33.74 ±0.75 | |  |
| **EDA** | 0.38 ±0.07 | 0.34 ±0.02 | | 0.39 ±0.05 | | 0.35 ±0.03 | | 0.38 ±0.03 | | 0.48 ±0.10 | | 0.43 ±0.12 | | **0.56*** ±0.05 | | 0.25 ±0.03 | | 0.33 ±0.02 | | 0.29 ±0.08 | | **0.43*** ±0.01 | |  |
| **DGLA** | 1.09 ±0.16 | 0.99 ±0.21 | | 1.14 ±0.19 | | 0.99 ±0.12 | | 0.73 ±0.05 | | **0.99*** ±0.21 | | 0.77 ±0.25 | | **1.05*** ±0.18 | | 1.15 ±0.08 | | **1.46*** ±0.23 | | 1.09 ±0.12 | | 1.18 ±0.06 | |  |
| **AA** | 9.96 ±1.09 | **7.93*** ±0.21 | | 9.91 ±1.77 | | **7.66*** ±0.27 | | 12.20 ±1.07 | | 13.09 ±0.70 | | 10.68 ±1.19 | | 10.81 ±0.41 | | 5.63* ±0.37 | | 4.74 ±0.19 | | 4.63 ±0.47 | | 4.51 ±0.23 | |  |
| **Adrenic acid** | 0.16 ±0.00 | 0.17 ±0.01 | | 0.18 ±0.04 | | 0.15 ±0.01 | | 0.49 ±0.04 | | **0.42*** ±0.03 | | 0.50 ±0.04 | | 0.48 ±0.04 | | 0.07 ±0.03 | | 0.05 ±0.01 | | 0.05 ±0.02 | | 0.04 ±0.01 | |  |
| **Osbond acid** | 0.14 ±0.00 | 0.18 ±0.03 | | 0.27 ±0.04 | | 0.23 ±0.02 | | 2.84 ±0.33 | | 2.93 ±0.41 | | 3.14 ±0.37 | | 3.23 ±0.54 | | 0.10 ±0.02 | | 0.08 ±0.03 | | 0.06 ±0.02 | | 0.09 ±0.01 | |  |
| **Omega-9 Fatty Acids** | | | | | | | | | | | | | | | | | | | | | | | | |
| **Oleic acid** | 6.99 ±0.56 | | 6.24 ±0.78 | | 7.43 ±0.42 | | **5.80*** ±0.22 | | 6.05 ±0.54 | | 5.85 ±0.44 | | 5.68 ±1.82 | | 5.62 ±0.92 | | 5.67 ±0.29 | | 5.87 ±0.47 | | 5.33 ±0.61 | | 4.99 ±0.45 | |
| **Gondoic acid** | 0.34 ±0.05 | | 0.45 ±0.11 | | 0.24 ±0.08 | | 0.27 ±0.02 | | 0.25 ±0.07 | | **0.41*** ±0.12 | | 0.27 ±0.16 | | **0.41*** ±0.07 | | 0.19 ±0.03 | | 0.26 ±0.02 | | 0.24 ±0.07 | | 0.38 ±0.13 | |
| **Nervonic acid** | 0.32 ±0.07 | | 0.25 ±0.04 | | 0.28 ±0.07 | | 0.24 ±0.01 | | 0.32 ±0.11 | | 0.26 ±0.03 | | 0.27 ±0.04 | | 0.27 ±0.09 | | 0.25 ±0.04 | | 0.24 ±0.04 | | 0.30 ±0.10 | | 0.30 ±0.04 | |

Abbreviations: ALA - α-linolenic acid, EPA - eicosapentaenoic acid, DPA - docosapentaenoic acid, DHA - docosahexaenoic acid, LA - linoleic acid, EDA - eicosadienoic acid, DGLA - dihomo-gamma-linolenic acid and AA - arachidonic acid.
