# Supplementary figures and images for: Vulnerability to omega-3 deprivation in a mouse model of NMDA receptor hypofunction
Source: NPJ Schizophr. 2017 Mar 22;3:12. doi: 10.1038/s41537-017-0014-8 (PMC5441542; doi:10.1038/s41537-017-0014-8)

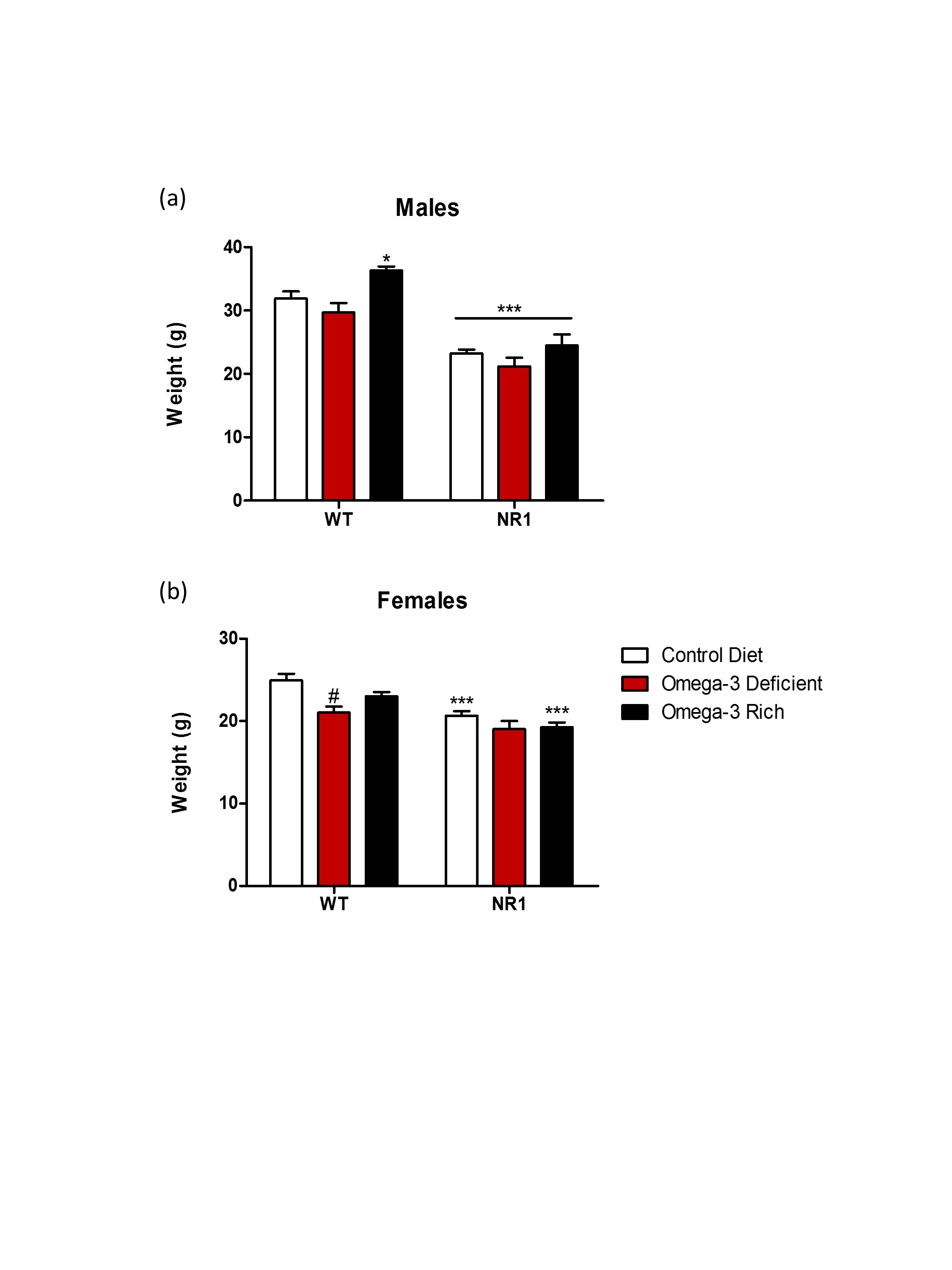

Supplement: Supplementary file 4 — Supplementary Figure 1 [file 41537_2017_14_MOESM4_ESM.jpg]

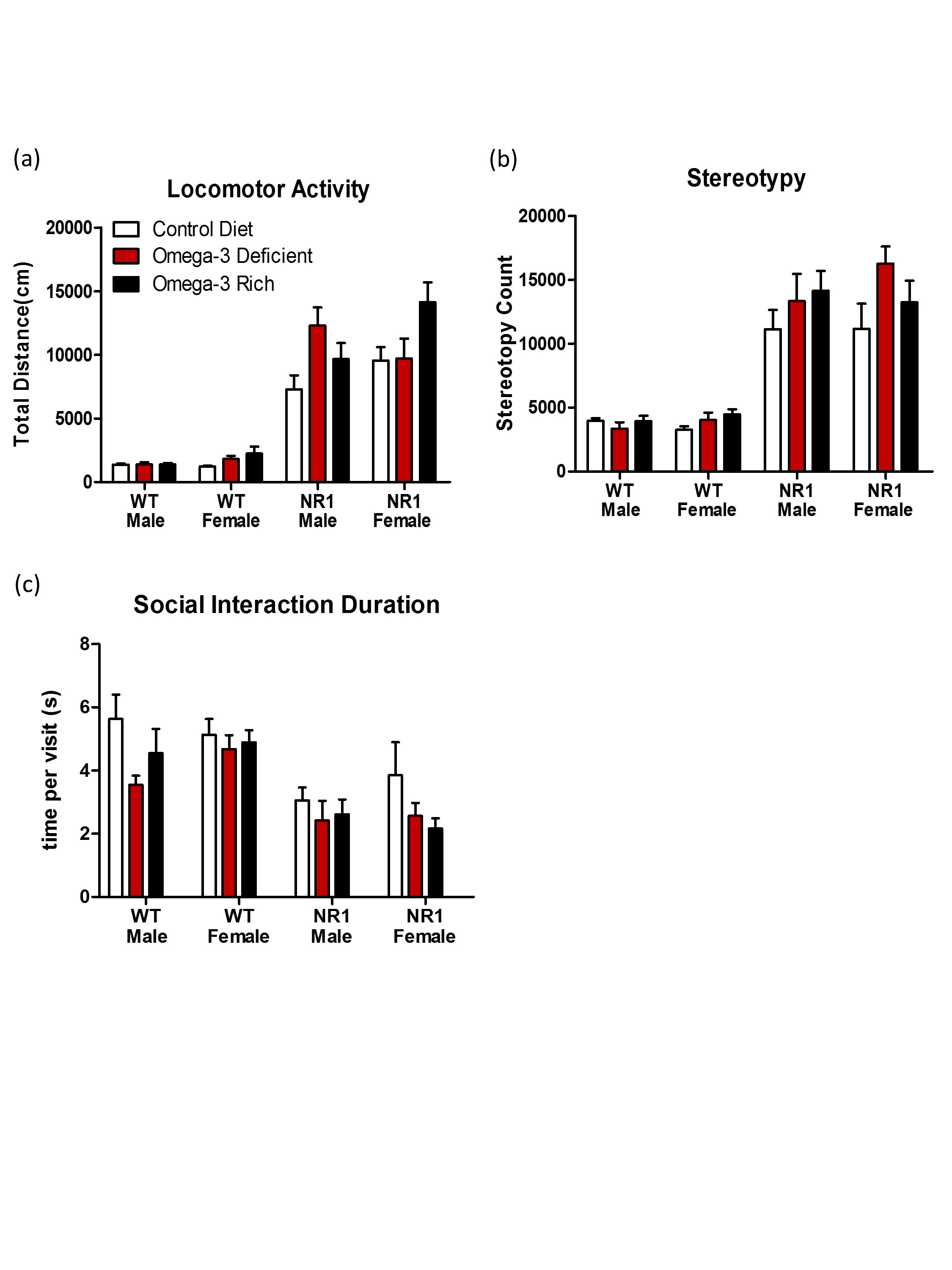

Supplement: Supplementary file 5 — Supplementary Figure 2 [file 41537_2017_14_MOESM5_ESM.jpg]

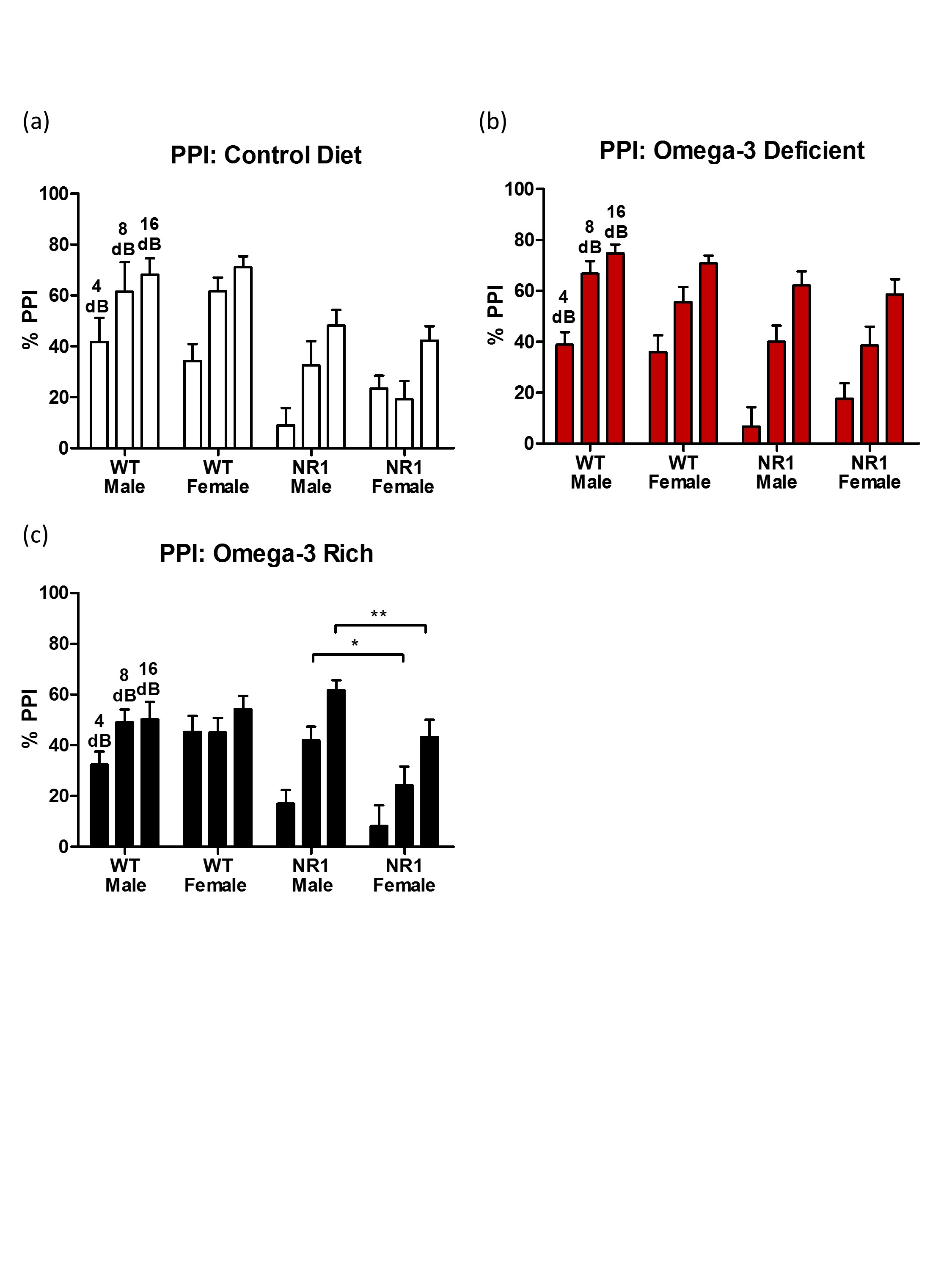

Supplement: Supplementary file 6 — Supplementary Figure 3 [file 41537_2017_14_MOESM6_ESM.jpg]

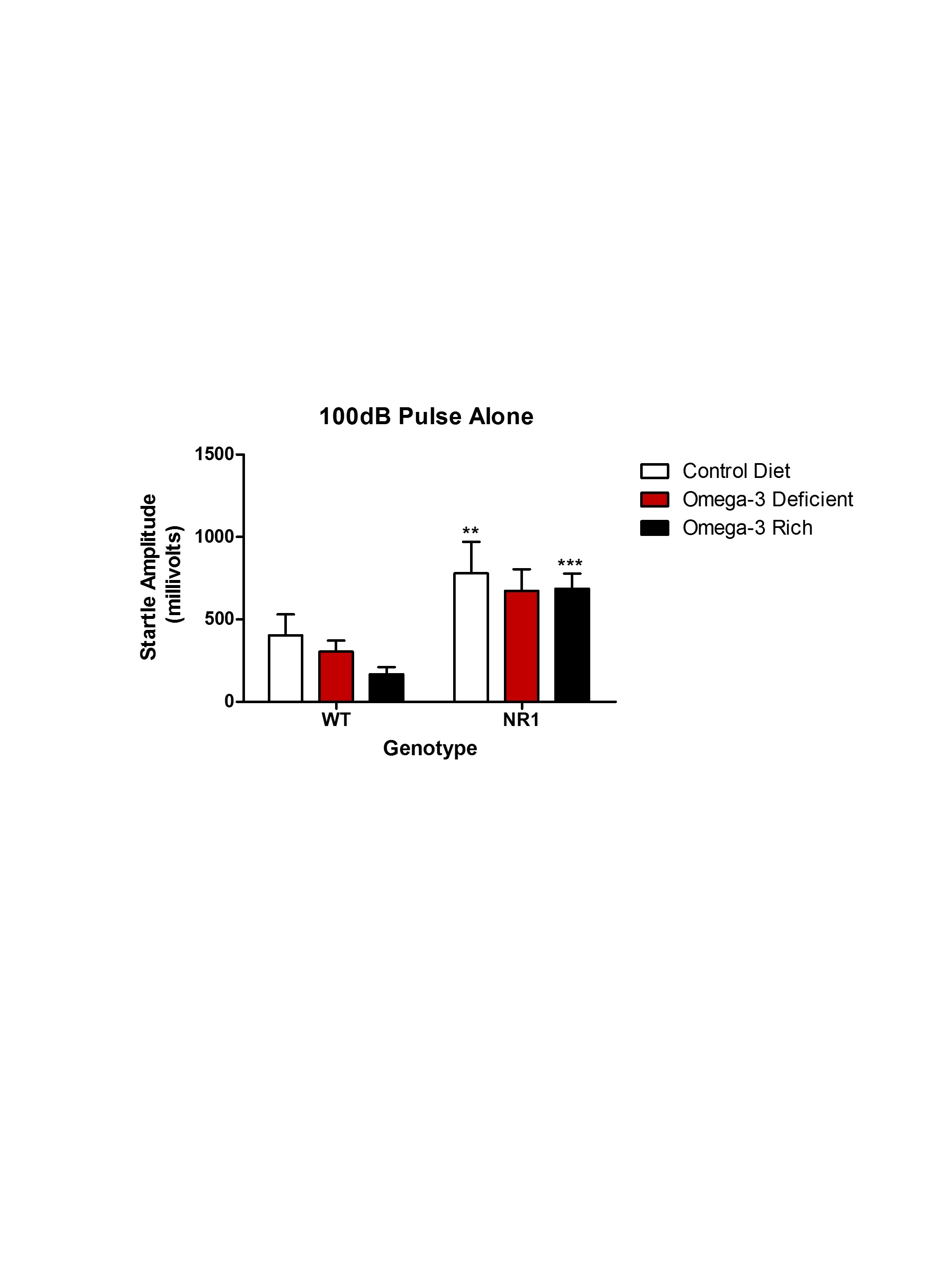

Supplement: Supplementary file 7 — Supplementary Figure 4 [file 41537_2017_14_MOESM7_ESM.jpg]

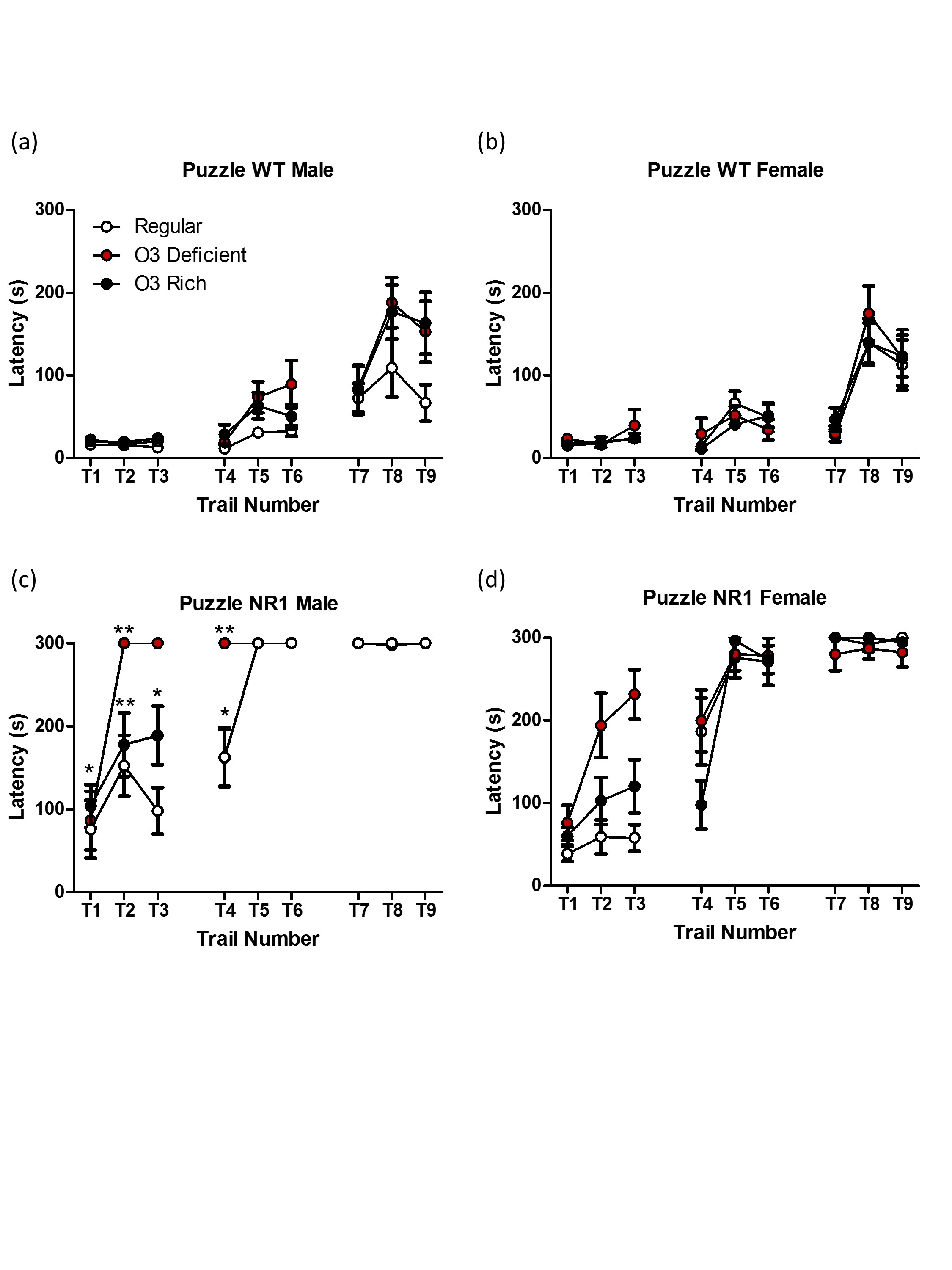

Supplement: Supplementary file 8 — Supplementary Figure 5 [file 41537_2017_14_MOESM8_ESM.jpg]
